# Effects of Inoculating the Lower Animals with Diphtheritic Exudation

**Published:** 1880-11

**Authors:** 


### EFFECTS OF INOCULATING THE LOWER ANIMALS WITH DIPHTHERITIC EXUDATION.

Supplement No. 7 of the *National Board of Health Bulletin* contains a detailed report concerning a series of experiments and observations on the above subject by Drs. H. C. Wood and Henry F. Formad, of Philadelphia.

These gentlemen inoculated eighteen rabbits, three dogs, ten cats, and one goat, with the membranous exudation from persons sick with diphtheria, by inserting it beneath the skin or into scarifications in the membrane lining the mouth, and in some by introducing it in both places. Of the thirty-two animals thus inoculated, only six died. In these six, the time between the inoculation and death varied from two to fifteen days.

In only one of the fatal cases was there found any false membrane on the mucous membrane of the fauces or larynx, and in that one it was very slight. Neither did these gentlemen find any micrococci, either in the internal organs of those which died or in the blood of the living.

The post-mortem examinations did show that "in every case the internal organs were tubercular, and in many cases intensely so." They also state explicitly that "in no case did inoculation in the mouth produce either local or general symptoms."

To determine whether the rapid development of tuberculosis in the animals inoculated was caused by any *specific* influence of the diphtheritic matter used, or only the result of a local inflammation from any cause, nine rabbits were treated by inserting simply small bits of wood or glass, or bit of wire, and in one a bunch of clean hair, beneath the skin. Five of the nine sickened and died at periods varying from fifteen to twenty-eight days. In each case the post mortem examinations showed extensive tubercular infiltrations in nearly all the internal organs, differing in no essential particular from the appearances in those dying after inoculation with the diphtheritic matter. In four rabbits the diphtheritic exudative matter was applied to the mucous membrane lining the trachea. In one of the four, pseudo-membranous trachitis was produced, followed by death; the other three remained well. The experimenters next tried the effects of applying simple irritants to the tracheal membrane. They applied a few drops of aqua ammonia in five rabbits, one cat and one dog. One of the rabbits died at once from the operation; too much ammonia having been used. Of the other six, five died in from one to three days, all presenting well formed pseudo-membranous formations in the larynx and trachea, identical in appearance with the ordinary croupous membrane, and containing plenty of bacteria. A fifth series of experiments consisted in the inoculation of the trachea in ten rabbits, with pus and other morbid animal substances. Of these, six died in periods ranging from one to nine days; only two of them presenting well marked false membrane in the trachea. It will be seen that the whole number of animals experimented on was sixty-two.

Drs. Wood and Formad close their interesting report with the following comments:

"In looking over the last table, it will be seen that in two of the ten experiments pseudo-membranous trachitis was caused by the introduction of organic matter into the trachea. In both of the cases in which false membrane was produced, the injected material was pus; and it will be noticed that only four such experiments were made, so that the proportion of successful result is very large; much larger, indeed, than with true diphtheritic exudation in our experiments.

"Trendelenburg found that not only ammonia, but also various other chemical irritants are capable of causing the formation of false membrane in the trachea; many years since it was proven that tincture of cantharides will do the same thing. It would seem, therefore, that in the trachea the formation of a pseudo-membrane is not the result of any peculiar or specific process, but simply of an intense inflammation, which may be produced by any irritant of sufficient power. This fact, certainly, is very suggestive in regard to the pathology of diphtheria, and whilst we are not prepared to commit ourselves to any theory, it does seem proper to call attention to certain facts as indicating a very simple explanation of the peculiarities of the disease.

"It is certain that as in the lower animals, so also in man, will chemical irritants produce a pseudo-membranous trachitis; we are also well assured that there is no anatomical difference which can be detected with the microscope, between the lesions of true croup and diphtheritic angina. A difference has been believed by some pathologists to exist between the two diseases, in that in croup the membrane separates easily; in diphtheria with great difficulty from mucous membrane. This seems to arise from a misunderstanding. The mucous membrane of the fauces and mouth has a squamous not easily detached epithelium, and consequently membrane connected with or springing from such surface is firmly adherent. The epithelium of the trachea is columnar, ciliated, and detaches with the utmost facility even in normal condition of the organ; hence membrane attached to it separates readily. The membrane of diphtheritic trachitis is always readily detached in the line of the epithelium. Our preparations also show that the exudation of the croupous inflammation excited artificially in the trachea is not merely superficial, but also extends below the basement membrane. Some of the best clinical authorities of the day teach that there is no essential clinical difference between true croup and diphtheria, cases commencing apparently as local sthenic inflammation and ending as the typical adynamic systemic poisoning. Every practitioner must have seen cases of angina in which he was in doubt whether to call the affection diphtheria or not; the very frequent diagnosis of "diphtheritic sore throat" is a strong evidence of this. There have been cases in which diphtheritic matters absorbed by a wound have produced symptoms very closely resembling those of ordinary septic blood poisoning from post mortem wounds, etc.; there have been cases of the formation of false membrane about wounds, etc., without any known exposure to a specific diphtheritic poisoning, indicating that the systemic tendency to this peculiar form of exudation is capable of being engendered by other than the specific poison of diphtheria; finally, diphtheria seems sometimes to be produced by exposure to cold.

A general view of these facts seems to indicate that the contagious material of diphtheria is really of the nature of a septic poison which is also locally very irritant to the mucous membrane; so that when brought in contact with the mucous membrane of the mouth and nose it produces an intense inflammation without absorption by a local action. Whilst absorption is not necessary for the production of the angina, it is very possible that the poison may act locally after absorption by being carried in the blood to the mucous membrane. Further, under this theory it is possible that the poison of diphtheria may cause an angina which shall remain a purely local disorder, no absorption occurring, or a simply local trachitis produced by exposure to cold or some other non-specific cause may produce the septic material when absorption shall cause blood poisoning, the case ending as one of adynamic diphtheria.

Some such an explanation as those here offered seems to reconcile antagonistic opinions concerning the value of local treatment in diphtheria; because it is plain that the value of such treatment must largely depend upon whether the angina has or has not been preceded by absorption.

There is one more important clinical feature of the disorder, which under other views of the disease seems inexplicable, but which with the present theory is easily explained. Diphtheria differs from the exanthemata by the fact that one attack in no way protects against a second. It will be seen that the theories here put forward remove the affection entirely from any relation with exanthema; placing it rather with septic diseases; which as is well known, may recur indefinitely.

We want, however, distinctly to state that we do not consider these ideas to be more than suggestions, and it is useless to speculate, except as a guide to further experimental research; it does seem to us that there are now two pathways clearly open which if carefully followed must lead to important positive or negative results. The first of these consists in the making of careful culture experiments to determine whether there is or is not any difference between the bacteria of ammonia and diphtheritic false membranes; the second, the study of the induction of epidemics of pseudo-membranous angina and trachitis in the lower animals and the relation to these of the rapid cases of death produced in the lower animals by diphtheritic inoculation.

There is still another somewhat different view which seems also not repugnant to the known facts of the case. There may be bacteria, which although they offer no points of difference detectable by our best microscopes, are really very diverse. Two spermatozoa or two ova in the higher animals may seem to be exactly alike and yet be potentially widely separated. Although therefore the bacteria of an ammonia false membrane seem identical with those of diphtheritic false membrane, they are not of necessity really so. Careful studies of the blood of patients who die of diphtheria should be made, but at present it seems altogether improbable that bacteria have any direct function in diphtheria; i. e., that they enter the system as bacteria and develop as such in the system and cause the symptoms. It is, however, possible that they may act upon the exudations of the trachea as the yeast plant acts upon sugar, and cause the production of a septic poison which differs from that of ordinary putrefaction, and bears such relations to the system as to, when absorbed, cause the systemic symptoms of diptheria. Now, these bacteria may be always in the air, but not in sufficient quantities to cause trachitis, but enough when lodged in the membrane to set up the peculiar fermentation ; whilst during an epidemic they may be sufficiently numerous to incite an inflammation in a previously healthy throat.

